# Detection of Dairy Herd Management Issues Using Fatty Acid Profiles Predicted by Mid-Infrared Spectrometry

**DOI:** 10.3390/ani15111575

**Published:** 2025-05-28

**Authors:** Sébastien Franceschini, Claire Fastré, Charles Nickmilder, Débora E. Santschi, Daniel Warner, Mazen Bahadi, Carlo Bertozzi, Didier Veselko, Frédéric Dehareng, Nicolas Gengler, Hélène Soyeurt

**Affiliations:** 1 TERRA Research and Teaching Centre, Gembloux Agro-Bio Tech, University of Liège, 5030 Gembloux, Belgium; sfranceschini@uliege.be (S.F.); claire.fastre@uliege.be (C.F.); charles.nickmilder@uliege.be (C.N.);; 2 Lactanet, Saint-Anne-de-Bellevue, QC H9X 3R4, Canada; 3 Walloon Breeders Association, 5590 Ciney, Belgium; 4 Comité du Lait, 4651 Herve, Belgium; 5 Walloon Agricultural Research Centre, 5030 Gembloux, Belgium

**Keywords:** fatty acids, FT-MIR spectrometry, unsupervised hierarchical clustering, dairy cows, herd management

## Abstract

This article focuses on the creation of a monitoring tool using routinely collected data from milk payment analyses. Milk samples were analyzed through Fourier Transform mid-infrared spectrometry every 1 to 3 days, and their compositions were predicted using machine learning models. Among the predicted parameters, fatty acid profiles appear to be effective indicators of animal status and management practices. In this research, these profiles were summarized using 31 fatty acids or groups of fatty acids. The methodology consists of four steps: hierarchical clustering to detect patterns in a Belgian spectral dataset (*N* = 774,781), interpretation of the identified seven clusters, development of predictive models applied to a Canadian dataset (*N* = 670,165), and validation using management information collected from Canadian farms. The identified clusters revealed significant relationships with feeding management strategies and temporal evolutions, highlighting the potential to develop automated alert systems that assist farmers and advisors in herd monitoring.

## Simple Summary

Farms generate increasing amounts of data each year. One example is the bulk tank milk composition, predicted through spectrometry, which is routinely measured for milk payment purposes. Among the different milk components, the fatty acid profile is of utmost importance because it is closely linked to animal status and farm management practices. This research aims to explore a novel application of these fatty acid profiles by developing a practical herd-monitoring tool for farmers and advisors. The methodology developed consists of an unsupervised learning method that identifies meaningful patterns in fatty acid profiles, combined with an expert-driven interpretation of those patterns. The analysis was performed using a Belgian bulk tank milk database. Seven distinct patterns were identified; among these, three were associated with good management practices, three indicated potential risks, and one highlighted a metabolic disorder probably related to management practices. Most of these patterns were also observed in a Canadian bulk tank milk dataset, demonstrating that the method is generalizable across different regions and farming conditions. Moreover, the probabilities associated with each pattern can serve as a reliable foundation for creating practical alerts to support on-farm decision making, thereby enhancing farm management and contributing positively to animal welfare.

## 1. Introduction

In 2023, the Food and Agriculture Organization (FAO) emphasized the critical role of milk in enhancing nutrition and health due to its nutritional composition and significance in global food systems [1]. Cow milk typically comprises 86.9% water, 4.6% lactose, 4.2% fat, 3.4% protein, 0.8% minerals, and 0.1% vitamins. Among the fat, 98% consists of triglycerides associated with fatty acids (FAs) [2]. FAs are essential since their composition directly impacts milk quality, influencing physical, nutritional, and technological properties important for human consumption and dairy processing [3]. Moreover, the milk FA profile is a valuable proxy of the cow’s metabolic status [4]. Although approximately 400 different FAs exist in milk, only 12 FAs constitute more than 1% each in milk fat [2,5].

**Figure 1:**
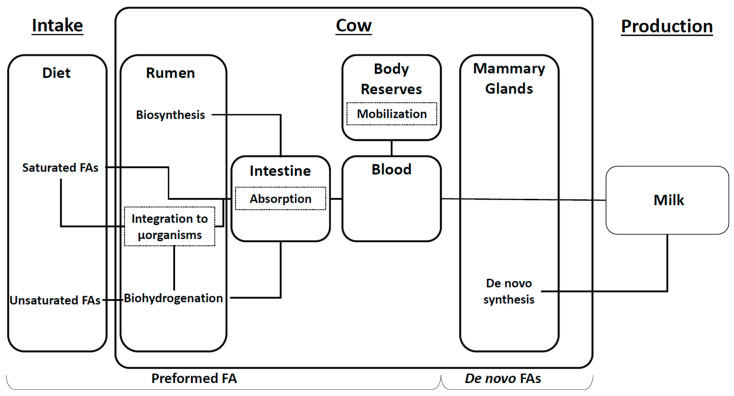
Diagram of fatty acid synthesis pathways (FAs: fatty acids, µorganism: microorganisms).

Based on their origins, FAs can be categorized into three main groups: preformed FAs (approximately 41%), mixed FAs (about 31%), and de novo FAs (around 28%). Understanding variation among these FA groups is crucial for evaluating the animal’s metabolic and nutritional status, since they originate from distinct metabolic pathways which are sensitive to management practices and health conditions. Preformed and de novo FA pathways are summarized in Figure 1.

De novo FAs are synthesized directly in the epithelial cells of the mammary gland from residues of ruminal microbial fermentation [3]. The main primers are acetate (two carbons) and butyrate (four carbons), onto which two-carbon molecules are successively added to produce FAs up to fifteen carbons long, which are immediately excreted in milk [6].

Preformed FAs originate from three sources: dietary intake, microbial FA synthesis in the rumen, and body fat mobilization. Dietary FAs, once digested and absorbed into the bloodstream, can enter milk unchanged or undergo microbial biohydrogenation in the rumen if polyunsaturated. Biohydrogenation saturates FA chains through microbial action in the rumen, forming intermediate unsaturated FAs before becoming fully saturated. Occasionally, intermediate products bypass the final saturation steps and are absorbed directly in their unsaturated form [7]. These ruminal processes greatly influence milk FA profiles and depend heavily on microbial populations present in the rumen.

These microbial populations can also synthesize FAs from ruminal fermentation residues [8]. The importance of each source depends on the microbial population and the proportion of lipids in the ration [9]. Biosynthesis mainly produces C16:0 and C18:0 from acetate and butyrate, but also odd- or branched-chain FAs from other primers. For example, using propionate (three carbons), microorganisms can synthesize odd-chain FAs, mainly C15:0 and C17:0. By using amino acid catabolism residues, the final products are branched-chain FAs [9]. These microbial FAs are then absorbed in the intestine, pass into the bloodstream, and are incorporated into milk fat.

The final source of preformed FAs is body fat, which can be mobilized by the animal. This source generally accounts for less than 10% of milk fatty acids, except after calving, when lactation begins and the animal’s energy deficit is greatest [10,11]. This deficit is due to the high energy demand for calving and the onset of lactation, as well as the reduced dry matter intake by the animal at this stage of lactation [12]. At the same time, the mammary gland loses its capacity to produce FAs de novo. To compensate for these phenomena, the animal will mobilize its body fat. In the first weeks of lactation, there are fewer de novo and more preformed FAs in the milk [13].

Those mentioned pathways are known to be influenced by many factors [2,11,14] such as breed and genetics [15]; herd management, including animal density and ration [16,17]; stage of lactation [13]; temperature [18,19]; animal health and physiology [20]; animal nutrition; and the main fermentation processes in the rumen, themselves linked to diet [21,22].

Thus, analyzing the changes in the FA profile of milk is a powerful tool to monitor a dairy herd routinely, whether to assess the impact of management practices or detect external factors impairing the herd, such as feed storage problems or heat stress events during periods of hot weather. However, a monitoring tool requires easy, cheap, and robust data acquisition. In this context, the Fourier Transform mid-infrared (FT-MIR) spectrometry already applied to milk bulk tanks for milk payment is a huge opportunity. FT-MIR spectrometry is a rapid and non-destructive method that can predict several key milk FAs accurately [15,23].

However, interpreting complex FA profiles routinely remains challenging due to the numerous FAs and their interactions. Traditional multivariate analyses provide global overviews but lack detail for specific patterns. Unsupervised machine learning techniques, specifically clustering and pattern recognition, may provide deeper insights into specific FA profile groupings, revealing less frequent yet significant patterns. These machine learning methodologies do not require a target to predict, which is often difficult to have at a large scale, but aim to find variables that are interacting together and that form groups of homogeneous observations. Another advantage of using an unsupervised approach is the possibility to recognize patterns on the entire spectral database and not only on a few records for which the target is observed, as is done when using supervised learning.

Recently, Franceschini et al. [24] demonstrated this by applying hierarchical clustering to FT-MIR predictions of individual cow milk samples. This approach was initially applied to individual cow milk samples from Dairy Herd Improvement (DHI) records using FT-MIR predictions related to animal health. In the present study, we extend this approach by utilizing spectral data obtained from milk analyses used for milk payment determination. Compared to DHI records, bulk tank milk records offer the advantage of higher sampling frequency (every 1 to 4 days) versus traditional milk recording (every 4 to 6 weeks). Furthermore, such data are available for all herds delivering milk to dairy processors, whereas DHI data are limited to herds enrolled in performance recording programs.

In conclusion, this study aims to address the complexity of interpreting the numerous FA profiles by identifying the most representative and informative ones, providing biological interpretation, and developing a predictive model. From those results, the final aim is to estimate the feasibility of establishing a decision support tool for dairy herd management, based on the FA profile of milk predicted by mid-infrared spectrometry.

## 2. Materials and Methods

**Figure 2:**
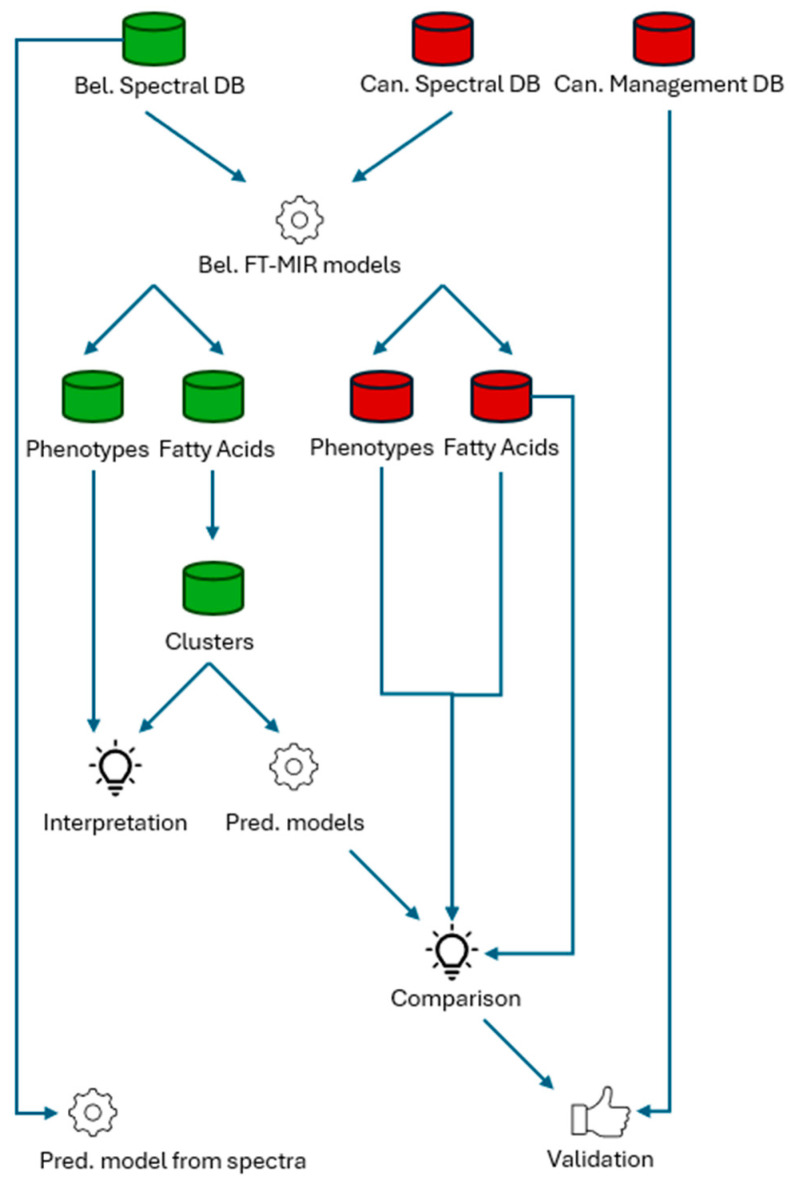
Workflow of the analysis. The colors indicate the origin of the dataset: green for Belgium and red for Canada. (Bel = Belgian, DB = database, Can = Canadian, FT-MIR = Fourier Transform mid-infrared, Pred = predictions).

The software used for data processing and analysis was the R language, version 4.2.3 [25]. The entire workflow from databases to results is summarized in Figure 2.

### 2.1. Belgian Dataset

The first database used in this study is linked to the analysis of milk samples collected within the milk payment scheme in the Southern Region of Belgium called Wallonia. Access to this database is governed by the “Futurospectre” agreement between ULiège—Gembloux Agro-Bio Tech (Gembloux, Belgium), the Walloon Research Centre (CRA-W, Gembloux, Belgium), the Walloon Breeding Association (AWé, Ciney, Belgium), and the milk laboratory Comité du Lait (Battice, Belgium). No ethical approval was needed, as the milk samples were collected during the milk payment carried out routinely.

The milk samples, taken every 1 to 3 days by the dairies from the tanks of the farms collected, were analyzed using FT-MIR spectrometry by the Comité du Lait (Battice, Belgium). All samples were analyzed with Foss MilkoScan spectrometers (Foss, Hillerod, Denmark). During this analysis, in addition to the fat and protein contents predicted by the spectrometer, spectra were also recorded in a database. The spectra were standardized using the method developed by Grelet et al. [26]. Different prediction equations from Grelet et al. [27] were then applied to these spectra to extend the number of phenotypes available. These correspond to 31 phenotypes relating to FAs or FA groups (g/dL milk), milk production (kg/day), estimated fat content (g/dL milk), β-hydroxybutyrate concentration in milk (BHB; log, mmol/L plasma), energy balance, protein efficiency, free FA in blood (FFA; log, µEq/L plasma), and dry matter intake (kg/day).

The predicted FA concentrations have been modified to be expressed as a ratio in g/100 g fat from the predicted fat content. This unit has the advantage of reducing correlation with milk production and fat content, better reflecting the importance of different metabolic pathways responsible for producing these milk compounds.

To isolate potential heat stress effects, temperature and humidity measurements were added to the database. The zip codes of Walloon municipalities were linked to the nearest meteorological station. The map of Belgian municipalities was superimposed on that of the 30 Walloon weather stations in the AGROMET network [28] using the Voronoi method as presented by Nickmilder et al. [29]. This method involves assigning to each station a polygon of a given area on the map. Municipalities covering more than one polygon were linked to the polygon on which most of their surface area was located. Finally, the temperature humidity index (*THI*) was calculated using the following formula:

(1) $$THI=\left(0.8\times tsa\right)+\left(\frac{hra}{100}\times \left(tsa-14.4\right)\right)+46.4$$

where *tsa* is the ambient dry temperature (°C) and *hra* is the ambient relative humidity (%) [30]. Thanks to this link between dairy records and weather stations, meteorological information could be merged with the spectral database.

Then, the resulting database was cleaned according to ICAR’s recommendations for recording of dairy cattle milk data [31]. These recommendations were created for the cow level, but it is expected to work at bulk tank milk level, as it is a weighted average of the herd. Only values between 1.5% and 9% fat and between 1% and 7% protein in g/dL of milk were retained. Rows containing outliers for fat and protein, or negative values for fatty acids or missing values for one of the traits studied, were discarded.

Then, the standardized Mahalanobis distance (GH) was calculated for each record to detect potential extreme data and compare the datasets from Belgium and Canada. To calculate it, it was necessary to perform a principal component analysis (PCA) on the 31 studied phenotypes, given the high correlations existing between some of them. Following this PCA, six principal components (PCs) were retained, as they explained 95.48% of the variability in the data. This analysis was performed using the FactoMineR package, version 2.4 [32]. Next, the *GH* distance was calculated as follows [33]:

(2) $$GH=({\left(\bar{x}-\bar{µ}\right)}^{T}S^{-1}\left(\bar{x}-\bar{µ}\right))/nPC$$

where $\bar{x}$ corresponds to the vector of PCs of the observation, $\bar{µ}$ is the vector containing the mean of each PC, *T* denotes the transpose, *S*^−1^ denotes the inverse of the variance–covariance matrix of the PCs selected by describing the matrix $\bar{x}$, and *nPC* is the number of PCs. No high extreme samples defined by a *GH* > 5 were observed, so we decided to keep all samples, as we wanted to observe samples with abnormal behaviors. The cleaned database included 774,781 records collected from 2835 Walloon farms between December 2018 and December 2021.

### 2.2. Canadian Dataset

The second dataset used in the present work comes from Canadian milk recording. Lactanet (Ste Anne de Bellevue, QC, Canada) provided access to spectral data from tank samples collected in Quebec from January 2020 to March 2022 from 4676 farms. The samples taken every 1 to 3 days from the tank were analyzed by Foss MilkScan spectrometers (Foss, Hillerod, Denmark). Canadian spectral data were used to predict FT-MIR phenotypes using the Belgian prediction models. Next, the predicted FT-MIR phenotypes were cleaned using the same methodology as the one applied for the Belgian dataset. Prior to any analysis, the Canadian data were projected onto the Belgian PCA used to detect outliers to ensure that their variability was indeed included in the Walloon variability and that the Walloon results are applicable.

The cleaned Canadian dataset contains 670,165 records. The estimated descriptive statistics of Belgian and Canadian datasets along with the performances of the prediction models are mentioned in Table 1. The differences observed are mostly related to the differences between a pasture-based system in Wallonia (Belgium) compared to a more productive system in Canada, where fat supplementation is more common [34].

In addition to milk composition information, Lactanet has made additional information available to better understand herd management. All of these data are average values per herd, calculated for the 12 months prior to April 2022, and are available for 3006 farms. These traits and their related descriptive statistics are presented in Table 2. The transition index corresponds to the difference between expected animal production, based on production in the previous lactation, and the projected production of these animals, based on the production in the first test. This index is used to evaluate the strategy implemented for cows during the transition period. The management index provides information at the herd level from the average animal’s environment and management effect [35]. The index at the animal level is based on the difference between the phenotype and the genetic effect.

Information on rations given in 2021 was also available for 540 herds for which Lactanet advisors provided feeding advice. This information includes the theoretical percentage of dry matter in the ration (49.97 ± 28.97) and the theoretical percentage of corn silage in the ration (22.85 ± 17.45). The latest dataset used, supplied by Lactanet, concerns the presence of ventilation in the barn. These data were collected by survey on 2113 farms and indicate the presence or absence of additional ventilation in summer (Yes = 75.96%, No = 21.44%, and NA = 2.60%).

### 2.3. Unsupervised Learning

Since many of the problems that can be detected are linked to multiple FA deviations, and a single FA irregularity can be associated with various problems, a multivariate approach was necessary for a more comprehensive understanding of the relationships between FAs, and consequently, to better assess the overall situation of the herd. Given the lack of diagnostic data for Walloon herds, the innovation of this work lies in studying the relationships between variables to identify groups of herds with distinct FA profiles and to explain them. This study extends the work of Franceschini et al. [24] on individual cow milk samples. Based on the predicted phenotypes, the literature, and the more extensive Canadian data, the identified clusters were interpreted, and their practical usefulness was assessed. From those results, the final objective was to study the feasibility of developing a specific decision-making tool, based on predicted FAs, to assist dairy farmers in their daily decision making.

### 2.4. Hierarchical Clustering

A hierarchical clustering algorithm was applied to the 31 Belgian FA predictions, with the aim of grouping together samples with similar characteristics. The function used was hclust from the Stats package version 3.4.1 [25]. This algorithm groups data according to their distance from each other. In the present study, the “ward.D2” method was used to calculate these distances between groups. The algorithm sums the squares of the distances between the data, then merges the data in such a way that the intra-group dispersion is minimized [36]. This method requires too much computer memory to be applied to the entire dataset. Therefore, a representative subset was created in 2 steps. Firstly, records with the greatest GH distances (GH ≥ 3) were kept, as they are the most extreme ones reflecting milk compositions different from the average. This represented 12,322 samples. Second, to equilibrate normal and more extreme samples, we selected 5000 samples randomly in every other GH strata (from 0 to 1, from 1 to 2, and from 2 to 3). So, the final subset was composed of 27,322 records. The number of clusters selected was a function of the cluster height, visible on the cluster dendrogram, as well as the distance differential with respect to the previous merge. The visualization of the first two PCs was used to illustrate the position of the various clusters. The 8 other indicators available in the database were added as additional features in this PCA to help the interpretation of these results.

### 2.5. Cluster Prediction

To facilitate the prediction of clusters in the available datasets, partial least squares discriminant analysis (PLS-DA) and random forest (RF) models were applied to the subset for which the cluster labels were known. The PLS-DA was estimated using the Caret package, version 6.0-90 [37], while the RF model was estimated using the randomForest package, version 4.7-1.1 [38]. PLS-DA was selected because some FA traits are correlated. The analysis was performed on 31 centered and scaled FAs, with a maximum of 30 components. The predicted cluster is the one with the highest probability of membership. A ten-fold cross-validation was used to determine the optimal number of PLS components and to assess the classification’s performance. Performance was measured using global accuracy and Cohen’s Kappa coefficient, which accounts for agreement occurring by chance. The random forest model was chosen to capture potential non-linear relationships. The Gini index was used as the splitting criterion, and the number of trees was set to 500. The maximum number of features was optimized through cross-validation, following the same methodology used for PLS-DA.

Since the 31 FAs were predicted by FT-MIR spectrometry, PLS-DA and RF models were also created, directly using the spectra to predict the clusters. This approach enhances the model’s transferability by eliminating the FA prediction step, which may vary depending on the lab or equation model used. A first derivative was applied to the spectra, and 212 spectral points were utilized as suggested by Grelet et al. [27]. The modeling methodology was the same as that used for the model based on the 31 FAs. After cross-validation, cluster predictions using PLS-DA and RF from FAs and from spectra were performed on all available Belgian and Canadian datasets.

### 2.6. Interpretation

To interpret the clusters, the means and standard deviations of each FA and other available sources of information were calculated according to the predicted clusters. This allowed us to compare the clusters.

The means associated with each cluster are essential for understanding the underlying patterns, but the frequency of each cluster and the frequency of transitions between clusters are also crucial for interpretation. For this reason, the transition matrix between clusters was estimated. For each observation in a specific cluster at time t, we measured the probabilities of transitioning to every cluster at time t + 1. In this approach, transitions between clusters were considered in a binary manner: from cluster 1 to 1, from 1 to 2, and so on. This allows for consideration of the dynamics between clusters.

Since the emergence of a problem in a herd is rarely spontaneous, the transition from one cluster to another may be too abrupt as a monitoring tool. Therefore, it is important to use quantitative rather than qualitative information. In this study, the probabilities of cluster membership obtained from PLS-DA and RF were also considered as a monitoring tool. To validate their usefulness, we calculated the correlations between cluster probabilities and FAs and indicators available in all databases. These probabilities were further used to visualize the evolution of herd conditions over time.

Finally, using additional validation data provided by Lactanet on animals, herd breakdown, and feeding, the herds found in each cluster were analyzed to determine whether it was possible to identify any trends in the type of animals or management. This was carried out to explain certain clusters, but also to confirm hypotheses about the herd status in the different clusters.

## 3. Results and Discussion

### 3.1. Clustering on Belgian Dataset

**Figure 3:**
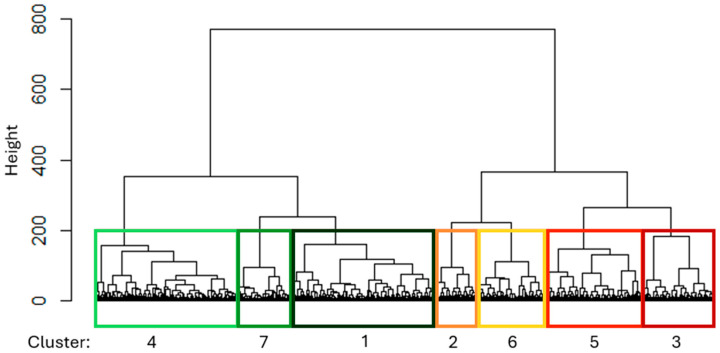
A dendrogram of the seven clusters found from the selected subset.

Seven clusters were retained after clustering the selected subset (*N* = 27,322) based on the separation observed in the dendrogram (Figure 3). The distribution of observations across the clusters is strongly influenced by the GH value (Table 3), confirming the hypothesis that rare observations with high GH values must be considered to understand the overall picture. Moreover, the less frequent clusters associated with high GH values could be linked to stress within the herd. Indeed, it is reasonable to assume that stressed herds would exhibit more extreme patterns with lower frequencies in the dataset. However, further analysis is necessary to confirm this hypothesis.

**Figure 4:**
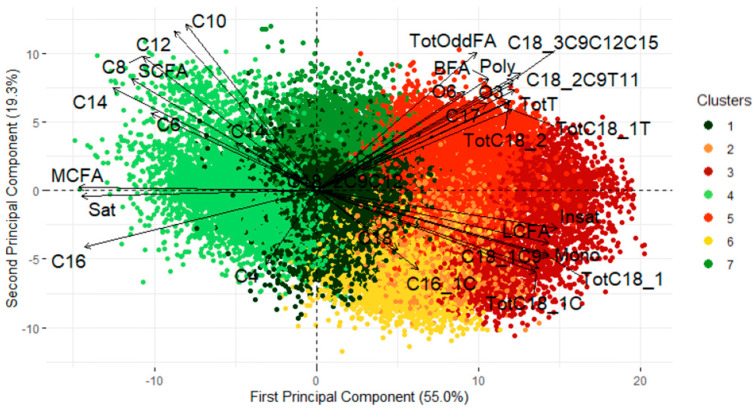
Projection of subset samples on the first two principal components (*N* = 27,322) (SCFA = short-chain fatty acids, MCFA = medium-chain fatty acids, LCFA = long-chain fatty acids, Sat = saturated fatty acids, Mono = monounsaturated fatty acids, Poly = polyunsaturated fatty acids, Insat = total of insaturated fatty acids, BFA = branched fatty acids, TotT = total of trans fatty acids).

The records for this subset were projected onto the first two PCs which together explained 74.3% of the whole dataset’s variance, as shown in Figure 4. The first axis is associated with milk saturation. The top-left quadrant corresponds to de novo FAs, except C4, which, along with C16, is associated with the bottom-left quadrant. The top-right quadrant is related to trans long-chain FAs, while the bottom-right is associated with cis long-chain FAs. The scatterplot illustrates an overlap among clusters with a visible transition from one cluster to another. This configuration was expected, as the underlying natural processes of metabolic issues and stress are continuous.

To facilitate cluster interpretation and to work on the complete dataset, supervised prediction models—PLS-DA and RF—were applied to the subset with known cluster labels. An advantage of this approach is its ability to estimate cluster membership probabilities, providing quantitative information that may be particularly valuable for decision support tools. In the past, Franceschini et al. [24] adopted a similar methodology.

For this study and based on the 31 FT-MIR-predicted FAs, a cross-validated PLS-DA predicted clusters with an average accuracy of 66.01% and a Cohen’s Kappa coefficient of 60.13%, using 16 components. On the same dataset, a cross-validated RF achieved an average accuracy of 91.81% and a Cohen’s Kappa coefficient of 90.14%. The same prediction methods (PLS-DA and RF) were applied directly to the standardized spectra to predict clusters, yielding relatively strong performance. The cross-validated global accuracies were 68.84% for PLS-DA with 29 components and 79% for the RF. A hypothesis about the differences in performance could be made regarding the non-linearity of the relation between some FAs and the clusters. The decrease in accuracy for RF when switching to spectra could be linked to the high dimensionality and the higher correlation between spectral wavelength absorption than between FAs.

For the following analysis of this paper, the RF model based on the 31 FAs was used to predict the cluster for the whole dataset, as it is the model with the highest global accuracy (91.81%), so most of the observations are correctly classified and misclassifications primarily occur in adjacent clusters, which can be explained by the cluster continuum shown in Figure 4 and Table 4.

### 3.2. Cluster Prediction on Belgian and Canadian Datasets

**Figure 5:**
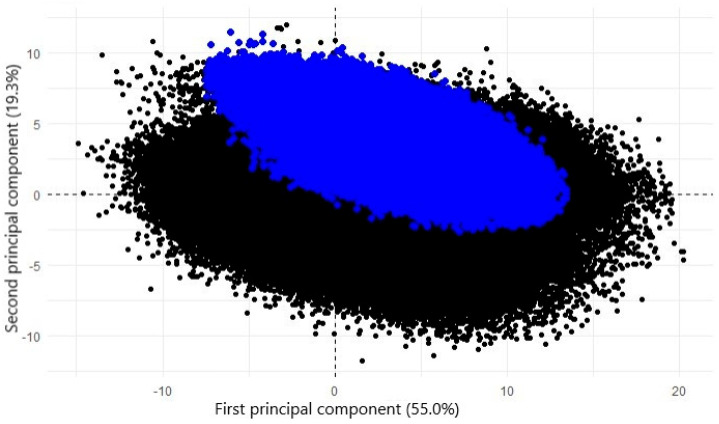
Projection of Canadian data (blue) onto graph of individuals (black) on first two principal components estimated based on whole Belgian database.

Before making any predictions using the Canadian dataset, it was essential to verify that the variability in the Canadian data is encompassed within the variability in the Belgian data used for clustering. Figure 5 illustrates the projection of the Canadian data onto the first two PCs initially developed from the Belgian database, confirming that the variability in the Canadian data is included within the Belgian FA data’s variability. Consequently, the RF model developed using the Belgian FA data can be applied to the Canadian data.

Using the developed RF algorithm, cluster membership was predicted for all Belgian (*N* = 774,781) and Canadian (*N* = 670,165) records. Table 5 presents the proportion of records by cluster for both countries, illustrating differences not only between Belgium and Canada but also compared to the GH (Table 4). We assumed that higher GH milk spectra are over-represented in the Belgian subset and that differences between countries may be due to the variability differences displayed in Figure 5.

First, there is minimal or no representation of Canadian data in Clusters 2, 3, 4, and 6. Second, the primary clusters in Belgium are Clusters 1 and 4, with Clusters 5 and 7 also occurring frequently, whereas Cluster 7 is predominant in Canada, with Clusters 1 and 5 following. One hypothesis, therefore, is that Clusters 1 and 4 represent an average situation in Belgium, while Cluster 7 represents an average situation in Canada. As most herds in a population are expected to be well managed, this would be the normal situation. At this step, more information such as the FA profiles is required to deepen interpretations.

To further explore the dynamics between clusters, transitions from one cluster to another were computed (Table 6). This table gives the proportion of records going from Cluster A at time t to Cluster B at time t + 1. In Belgium, Clusters 1, 4, and 5 have the highest proportion—around 65%—of successive records remaining within the same cluster. Clusters 1 and 4 appear to be interrelated, as approximately 20% of the records transition between them. A significant proportion of records from Cluster 5 moves to Cluster 1. In contrast, only a small proportion of records from Clusters 1 or 4 transit to Cluster 5. Instead, records in Cluster 5 predominantly come from Clusters 2, 3, and 6. Cluster 7 is less stable and less represented than Clusters 1 and 4, but it seems to be more related to these clusters than to others. In contrast, Cluster 3 rarely transits to Clusters 1 or 4.

Finally, Clusters 2 and 6 are the least stable, likely associated with temporary events. This could suggest that herds in Cluster 3 have experienced a deterioration in animal welfare, with herds remaining in this cluster longer than in others. This may indicate a problematic state and greater difficulty returning to a normal status. There are more transitions to Cluster 7 from Clusters 1 and 4, which are considered healthy clusters, than from Clusters 2, 3, 5, and 6, which are considered problematic. These observations suggest that Clusters 1 and 4 represent typical conditions in Wallonia, with Clusters 5 and 7 as intermediate clusters, and Clusters 2, 3, and 6 as potential problem clusters related to stress. If these hypotheses are correct and these clusters indeed reflect stress states, this dynamic would be expected.

In Canada, Cluster 7, which seems to be the standard, is also related to Clusters 1 and 4, even though Cluster 4 is not very represented in this dataset. Cluster 5 observations mostly stay in Cluster 5, and observations from Clusters 2, 3 and 6 go back to Cluster 5.

### 3.3. Cluster Interpretation

To deepen the interpretation of clusters, the means by cluster were calculated for phenotypes predicted by FT-MIR from bulk tank milk collected in Belgium and Canada (Table 7). To facilitate reading of the results, a color code was applied. The best and second-best values are in green and those with the worst values are in red. Globally, the results are similar for Belgium and Canada. The major differences between Belgium and Canada for FAs concern C18, polyunsaturated FAs, branched FAs, and odd FAs. For the supplementary variables, the protein, protein efficiency, energy balance, and blood free FAs are globally lower in Canada while the milk yield and the dry matter intake are higher. However, the performance of the equation for energy balance and blood free FAs is low, so the differences might be irrelevant. The maximum fat is also higher in Canada. Generally, Cluster 4 presents the best values in Wallonia followed by de-novo-FA-dependent Clusters 7 and Cluster 1, which have good values on average. Cluster 3 is the worst, followed by Cluster 6 and Cluster 2. Like Cluster 1, in terms of the “healthy” clusters, Cluster 5 seems to be intermediate. The same trends are observed for Canada. However, Clusters 2, 3, 4, and 6 are not very well represented in Canada. In a more controlled production and management system, these clusters may occur very rarely but may indicate more severe cases. Out of Clusters 1, 5, and 7, which represent 99.2% of the population, Cluster 5 would be the problematic cluster, with a potential transition to severe clusters.

So, Cluster 1 is the most represented in Belgium, followed by Cluster 4. These are the “standards” with better values for Cluster 4. Cluster 7, the most represented in Canada, has also good overall values. The difference in “standard” clusters between Canada and Belgium, and the small representation of some clusters, can probably be explained by the differences in farming systems between the two regions. Indeed, Quebec has few farms practicing grazing, unlike Wallonia. In Quebec, most farms use stanchion barns for lactating cows, whereas Walloon farms generally use loose housing when the animals are not in pasture [39,40]. The herd size in Quebec is 73 cows, and each animal gives an average of over 9300 L of milk per year [41] In Wallonia, there are 64 cows per herd, and the milk production is only 6600 L per year on average [42]. Moreover, those clusters are probably related to different types of feeding management. The interpretation is complex because many factors such as the type of diet, amount of concentrates, and fat supplementation are interacting with each other. The hypothesis is that Clusters 1, 4, and 7 correspond respectively to fresh grass, grass silage, and maize silage, potentially with concentrates and fat supplementation. Indeed, the grass silage diet is associated with higher C14 and C16 and lower C18 mono- and polyunsaturated FAs. The maize silage diet tends to increase the C6, C8, C10, and C12 found in Cluster 7 while the fresh grass diet favors an increase in C4 and C18:1*cis*9 [43]. Among the “healthy” clusters, Cluster 1 has the highest values for those traits. Concentrates are potentially found in Clusters 4 and 7 because they increase the percentages of C10 and C12 but also trans monounsaturated FAs and C18:2*cis*9*cis*12 [43]. Fat supplementation differs depending on the type of lipid. However, it is associated with an increase in polyunsaturated FAs and an inhibition of de novo FAs [43], which can explain the difference between Wallonia and Quebec for PUFAs and the inversion of the cluster order for de novo FAs between Clusters 4 and 7.

The hypothesis for Clusters 2, 3, 5, and 6 is that they include herds in a state of stress or poor health. Indeed, these clusters generally present the worst values for at least five traits. However, there are differences between these four clusters. First, Cluster 3 is the worst cluster, and is associated with extreme values for most of the traits. It is difficult to interpret the exact issue, as all the traits are extreme, but it is probably related to energy balance or ruminal acidosis. Moreover, Cluster 3 also presents a low value for the predicted milk yield.

Cluster 1, which is considered related to pasture or fresh grass, is similar to Clusters 2 and 6. However, Cluster 1 presents good values, unlike Clusters 2 and 6. After Cluster 3, Cluster 6 has the worse values for short-chain FAs, C16:1, and protein, and high values of THI. The low value for de novo FAs could indicate that there is an issue with rumen health, but for rare events, involving around 2.5% of the observations, such as dietary imbalance. Cluster 5, which has low values for C16 and high values for branched FAs, trans FAs, and odd-chain FAs, is also probably related to rumen health affected by the feeding strategy, such as being given a high-concentrate diet. This cluster is more common, involving 10% of the observations, and has low values for milk yield and energy balance, so it could be a combination with an energy balance issue. For those two clusters, farmers should monitor rumen health and adapt consequently. Cluster 2 has low values for fat, protein, unsaturated FAs, C14, C14:1c9, and DMI. It has also high values for blood free FAs and for long-chain FAs, more specifically C18 and C18:1c9, which is a sign of fat mobilization and negative energy balance. These clusters are essential, as they are transitioning to Cluster 3, which is an abnormal herd status. At this step, on-farm information is required to obtain a deeper interpretation.

Even if we have a high percentage of missing information, increasing the difficulty of drawing general conclusions, the management indicators provided by Lactanet should be useful information to further understand the clusters. Table 8 shows a higher percentage of organic farms and fewer herds with additional ventilation in Clusters 2 and 6 than in Clusters 1, 3, and 5. There is a significant difference between clusters in feed intake, with a very low proportion of corn silage in the rations of Clusters 2 and 3 (Table 9).

Table 10 shows herd indicators related to management, production, reproduction, and sanitary status averaged over twelve months. More than 50% of the Canadian herds in each cluster had data available for these different indicators. Cluster 6 was not found in this dataset. The data for Clusters 1 and 5 are very close or identical for most indicators, and they present the best values. Clusters 4 and 7 also had close, but worse values.

If we look at the transition index of these clusters, which represents the average herd’s performance at the start of lactation compared with what was expected of it, we observe that Clusters 2 and 3 have the lowest values, with negative values for Cluster 3. Cluster 5 is also slightly lower than the others. In other words, these herds showed lower performance in the first lactation than expected, suggesting a bad transition strategy between dry-off and early lactation. The milk management index, which estimates the environmental effect, shows negative values, but with very large standard deviations for all clusters. These indices are less negative for Clusters 1, 4, and 7. This trend is also found for the fat management index, suggesting that Clusters 2, 3, and 5 represent undesirable situations in Canada.

### 3.4. From Clusters to Probabilities

We showed that the clusters are related to herd management and could be of interest as a monitoring tool. However, we can improve the quality of the information given by using the probability of belonging to a cluster instead of a binary value. These probabilities are already a by-product of the RF algorithm, if we consider the proportions of trees that predict an observation into each cluster. These proportions respect the properties of probability. While these probabilities are correlated, which is expected, as the clusters overlap in Figure 4 and the underlying biological process is not discrete, the information they provide is useful for developing a monitoring tool on a routine basis. This information is mainly related to feeding management practices at the herd level and is available every one to three days. The FT-MIR predictions of the clusters and their probabilities become time series where extreme events can be detected and sent to the farmer.

**Figure 6:**
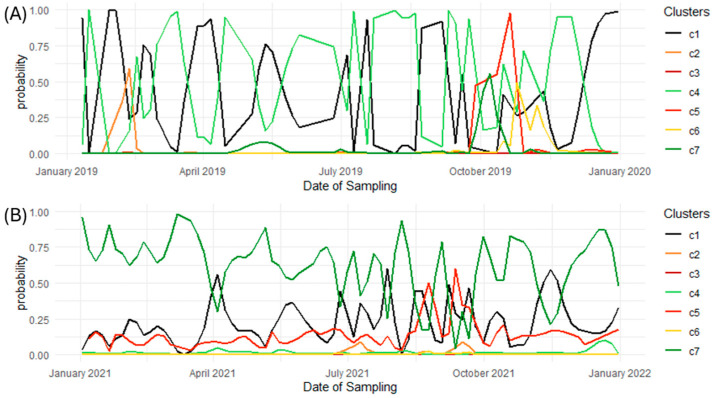
Evolution of cluster probabilities in time series for one farm in Belgium (**A**) and one farm in Canada (**B**).

An example of such a time series is represented in Figure 6. Every probability varies in time, and the switches between clusters happen when the cluster with the highest probability changes. This is typically the case when a peak occurs for specific issues. However, the peak is rarely based on one milk sample, so the increase in probability could be an early method of detecting management issues. In Belgium, the most observed clusters are Clusters 1 and 4, with some potential issues detected with Clusters 2 and 5. For Canada, it is mostly Cluster 7, then Cluster 1 and Cluster 5 for some potential issues. Those probabilities could be the sources of feedback for management.

### 3.5. Practical Implications and Study Limitations

The findings of this study suggest that FA profiles, summarized through a clustering approach, could be implemented as a herd-level monitoring tool for farmers. This tool would function as an alert system integrated into herd-monitoring software, where an alert would indicate the detection of an anomaly without necessarily identifying a precise cause. The alert system would not replace an expert opinion but could encourage the farmers to ask for help. Based on the cluster interpretation in this study, it appears that, even though the standard clusters differed between Belgium and Canada, likely due to different farming systems, Cluster 3 was consistently associated with abnormal FA profiles in both countries, even if Cluster 3 is not common in Canada. In Belgium, the observed dynamics between clusters showed transitions from intermediate clusters to Cluster 3, suggesting that monitoring the probability of belonging to these intermediate clusters could be used to detect early warning signals and mitigate health issues before escalation. The simplest solution would be to consider the cluster membership. So, Cluster 3 could be a red flag, while Cluster 2, 5, and 6 could be orange flags, and the others green flags. The red flag, which is severe, suggests that the farmer should contact an advisor or a veterinarian, while an orange flag suggests that they should evaluate the feed storage, the diet composition, or the fat supplementation, or check for a heat stress event and check the farm mitigation system.

However, the study presents several limitations. First, the interpretation of clusters remains complex due to the numerous interactions between FA profiles and management practices. Second, cluster validation is required using on-farm data and real-world health observations. While pattern detection from large FT-MIR prediction databases is a powerful tool, it cannot replace validation using reference measurements and expert field knowledge. Third, the alert methodology itself must be further fine-tuned to optimize the signal derived from the time series data. Fourth, practical deployment of such a system depends on a data infrastructure capable of delivering alerts rapidly enough to inform timely decision making at the farm level.

These limitations could be addressed through pilot trials on commercial farms, where feedback from farmers is available when the clustering approach flags potential issues. Such trials would support better interpretation of the clusters, confirm their utility, fine-tune the alert system, and help develop an experimental real-time data pipeline that could later be scaled for routine use. This would also allow for a first use case and for an assessment of its usefulness.

## 4. Conclusions

This study extends a previously developed clustering methodology—originally designed for individual milk samples—to bulk tank milk samples. The results suggest that the FT-MIR variability in Belgian herds encompasses that of Canadian herds, potentially reflecting a broader range of feeding systems in Belgium. Our findings indicate that this methodology could serve as a cost-effective tool for generating actionable indicators from existing milk payment data without imposing additional burdens on farmers. Notably, the transition from intermediate clusters to Cluster 3 appears promising as a reliable health-monitoring indicator. Integrating this approach into routine herd management may enhance the capacity to respond proactively to management issues. The final automated alert system should be merged with an individual level alert to improve the feedback given to farmers. However, field validation is required before widespread implementation, particularly to adapt the approach across diverse farming systems and geographical contexts.

## Figures and Tables

**Table 1:** Descriptive statistics for the 39 FT-MIR-predicted traits used in the study calculated from the available Belgian and Canadian spectra.

FT-MIR Predicted Traits ^1^	Unit	Belgium (*N* = 774,781)	Canada (*N* = 670,165)	R2cv	RMSE
Fat	g/dL of milk	4.17 ± 0.34	4.14 ± 0.27	/	/
Protein	g/dL of milk	3.47 ± 0.19	2.62 ± 0.13	/	/
Milk yield	kg/day	26.85 ± 2.85	29.97 ± 1.54	0.69	3.48
Energy balance		−3.12 ± 3.36	−7.05 ± 1.56	0.43	1.33
Nitrogen efficiency		53.67 ± 14.12	17.54 ± 1.25	0.52	1.44
Blood BHB	mmol/L plasma (log)	−0.82 ± 0.09	−0.78 ± 0.06	0.7	1.85
Blood free FA	µeq/L of plasma	495.15 ± 131.57	421.89 ± 84.79	0.39	344.2
Dry matter intake	kg/day	22.81 ± 2.14	24.18 ± 1.41	0.45	1.35
**C4**	**g/100 g of fat**	**2.68 ± 0.19**	**2.63 ± 0.13**	0.93	0.008
**C6**	**g/100 g of fat**	**1.81 ± 0.12**	**1.83 ± 0.08**	0.91	0.006
**C8**	**g/100 g of fat**	**1.18 ± 0.10**	**1.28 ± 0.07**	0.91	0.004
**C10**	**g/100 g of fat**	**2.65 ± 0.36**	**3.26 ± 0.25**	0.92	0.01
**C12**	**g/100 g of fat**	**3.32 ± 0.44**	**4.09 ± 0.33**	0.93	0.011
**C14**	**g/100 g of fat**	**11.43 ± 0.87**	**12.32 ± 0.65**	0.94	0.03
**C14:1*cis*9**	**g/100 g of fat**	**1.06 ± 0.11**	**1.17 ± 0.08**	0.71	0.008
**C16**	**g/100 g of fat**	**31.33 ± 3.25**	**28.90 ± 1.52**	0.95	0.091
**C16:1**	**g/100 g of fat**	**1.61 ± 0.17**	**1.56 ± 0.09**	0.73	0.013
**C17**	**g/100 g of fat**	**0.64 ± 0.05**	**0.66 ± 0.02**	0.81	0.003
**C18**	**g/100 g of fat**	**9.53 ± 1.03**	**9.05 ± 0.61**	0.84	0.056
**Total C18:1*trans***	**g/100 g of fat**	**3.12 ± 0.75**	**3.62 ± 0.37**	0.8	0.025
**C18:1*cis9***	**g/100 g of fat**	**18.56 ± 2.63**	**19.98 ± 1.74**	0.95	0.063
**Total C18:1*cis***	**g/100 g of fat**	**20.04 ± 2.78**	**21.57 ± 1.86**	0.95	0.061
**Total C18:2**	**g/100 g of fat**	**2.10 ± 0.22**	**2.54 ± 0.12**	0.71	0.014
**C18:2*cis9cis12***	**g/100 g of fat**	**1.25 ± 0.15**	**1.49 ± 0.11**	0.75	0.011
**C18:2*cis9trans11***	**g/100 g of fat**	**0.47 ± 0.10**	**0.61 ± 0.05**	0.74	0.01
**C18:3*cis9cis12cis15***	**g/100 g of fat**	**0.76 ± 0.33**	**0.96 ± 0.13**	0.69	0.004
**Saturated FAs**	**g/100 g of fat**	**68.49 ± 4.09**	**66.88 ± 2.06**	0.99	0.072
**Monounsaturated FAs**	**g/100 g of fat**	**26.78 ± 3.11**	**27.51 ± 1.97**	0.97	0.059
**Polyunsaturated FAs**	**g/100 g of fat**	**3.46 ± 0.68**	**4.44 ± 0.28**	0.79	0.021
**Unsaturated FAs**	**g/100 g of fat**	**30.33 ± 3.57**	**31.87 ± 2.15**	0.97	0.064
**Short-chain FAs**	**g/100 g of fat**	**8.77 ± 0.60**	**9.32 ± 0.42**	0.93	0.025
**Medium-chain FAs**	**g/100 g of fat**	**51.68 ± 3.95**	**51.89 ± 2.5**	0.97	0.104
**Long-chain FAs**	**g/100 g of fat**	**38.50 ± 4.12**	**39.04 ± 2.72**	0.95	0.11
**Branched FAs**	**g/100 g of fat**	**2.27 ± 0.26**	**2.67 ± 0.08**	0.77	0.013
**Omega3**	**g/100 g of fat**	**0.58 ± 0.12**	**0.71 ± 0.05**	0.68	0.006
**Omega6**	**g/100 g of fat**	**2.13 ± 0.24**	**2.58 ± 0.14**	0.74	0.014
**Odd-chain FAs**	**g/100 g of fat**	**3.82 ± 0.35**	**4.30 ± 0.12**	0.84	0.016
**Total Trans FAs**	**g/100 g of fat**	**3.91 ± 0.93**	**4.59 ± 0.46**	0.82	0.029
**Total C18:1**	**g/100 g of fat**	**23.15 ± 3.08**	**23.7 ± 1.99**	0.96	0.06

^1^ Traits in bold will be used in the following unsupervised analysis. FAs = fatty acids, BHB = beta-hydroxybutyrate.

**Table 2:** Herd characteristics for 3006 Canadian farms.

	Unit	Mean ± SD
Number of lactation cows	Cows	66.65 ± 50.87
Days in milk	days	176.99 ± 23.64
Margin on feed costs	$CA/cow/year	5009.77 ± 1254.85
Margin on feed costs per kg of fat	$CA/cow/year/kg	12.53 ± 1.52
Milk yield	L/day	26.59 ± 5.05
Fat	kg/cow/day	1.06 ± 0.30
Protein	kg/cow/day	0.86 ± 0.24
Milk production at lactation peak	L/day	39.72 ± 5.76
Days in milk at lactation peak	days	44.75 ± 4.54
Somatic cells in milk	×10^3^ cells/mL	182.02 ± 116.21
% of cows in the herd with somatic cells count > 200,000 cells/mL	%	18.95 ± 7.92
Urea in milk	g/mL	5.01 ± 5.90
% of cows in the herd with a urea concentration < 5 or >12	%	7.75 ± 9.90
Transition index		236.12 ± 445.85
% of cows in the herd with a negative transition index	%	37.98 ± 18.25
Age at first calving	month	25.30 ± 2.28
Calving interval	days	409.75 ± 31.72
Management index		−303.55 ± 1218.80
Management index for fat		−14.99 ± 52.65
% of the 3006 farms in conventional farming system	%	60.98
% of the 3006 farms in organic farming system	%	13.11
% of the 3006 farms with no information about their farming system	%	25.91

**Table 3:** Proportions of the seven clusters for the observations from the subset (*N* = 27,322).

	Cluster						
	Cluster 1	Cluster 2	Cluster 3	Cluster 4	Cluster 5	Cluster 6	Cluster 7
All data	23.55	7.90	11.72	22.75	15.89	10.81	8.71
GH < 3	27.53	2.29	4.07	28.51	15.64	5.48	13.47
GH > 3	17.98	12.51	21.04	15.73	12.54	17.29	2.91

**Table 4:** Confusion matrix related to random forest algorithm applied on 31 predicted fatty acids (*N* = 27,322).

		Reference						
		Cluster 1	Cluster 2	Cluster 3	Cluster 4	Cluster 5	Cluster 6	Cluster 7
Prediction	Cluster 1	5781	122	0	279	100	113	59
	Cluster 2	78	1651	24	0	30	57	0
	Cluster 3	0	43	2979	0	105	72	0
	Cluster 4	287	0	0	5875	0	36	46
	Cluster 5	50	36	111	1	4001	48	62
	Cluster 6	96	33	90	36	46	2623	8
	Cluster 7	53	0	0	24	59	4	2204

**Table 5:** Percentages of Belgian and Canadian records by cluster.

	Cluster 1	Cluster 2	Cluster 3	Cluster 4	Cluster 5	Cluster 6	Cluster 7
Belgium	38.47	1.18	1.04	36.60	10.40	2.51	9.80
Canada	12.00	0.28	0.04	0.48	13.22	<0.01	73.98

**Table 6:** Cluster transitions between 2 successive controls, expressed in %, for data collected in Belgium and Canada ^1^.

		Cluster 1	Cluster 2	Cluster 3	Cluster 4	Cluster 5	Cluster 6	Cluster 7
Belgium	Cluster 1	65.63	1.26	0.09	20.19	5.97	1.69	5.17
	Cluster 2	40.24	32.52	2.32	4.97	10.86	8.90	0.20
	Cluster 3	2.78	2.85	55.75	0.45	23.67	14.33	0.17
	Cluster 4	22.02	0.14	0.01	69.81	0.43	0.53	7.06
	Cluster 5	20.68	1.23	2.25	1.62	64.65	3.00	6.56
	Cluster 6	24.01	4.21	6.55	8.02	13.18	43.42	0.62
	Cluster 7	19.83	0.02	0.01	27.80	5.54	0.12	46.68
Canada	Cluster 1	34.39	0.28	0.01	0.31	11.10	0.00	53.92
	**Cluster 2**	**11.51**	**18.63**	**0.70**	**0.00**	**60.87**	**0.00**	**8.30**
	**Cluster 3**	**0.83**	**7.44**	**23.55**	**0.00**	**62.81**	**0.00**	**5.37**
	**Cluster 4**	**7.10**	**0.00**	**0.00**	**35.09**	**0.69**	**0.00**	**57.12**
	Cluster 5	10.27	1.24	0.17	0.02	63.08	0.00	25.22
	**Cluster 6**	**0.00**	**0.00**	**0.00**	**0.00**	**83.33**	**0.00**	**16.67**
	Cluster 7	8.79	0.04	0.00	0.38	4.43	0.00	86.35

^1^ Each line is first defined by a cluster at time t, and then the other columns represent the probabilities of moving into the cluster at time t + 1. Clusters in bold represent less than 1% of the dataset.

**Table 7:** Means by cluster of the phenotypes predicted by FT-MIR from bulk tank milk collected in Belgium and Canada as well as temperature humidity index (THI) for Belgium. Two best values are highlighted in shades of green, and worst in shades of red according to their rankings. Clusters for Quebec, in bold, represent less than 1% each.

	Belgium							Canada						
	C1	C2	C3	C4	C5	C6	C7	C1	**C2**	**C3**	**C4**	C5	**C6**	C7
C4	2.78	2.87	2.49	2.69	2.55	2.60	2.50	2.79	**2.85**	**2.63**	**2.75**	2.64	**2.55**	2.61
C6	1.82	1.7	1.46	1.86	1.70	1.56	1.84	1.80	**1.68**	**1.53**	**1.97**	1.74	**1.53**	1.85
C8	1.16	1.01	0.88	1.23	1.11	0.94	1.26	1.22	**1.08**	**0.97**	**1.39**	1.18	**0.95**	1.30
C10	2.53	2.06	1.78	2.77	2.51	1.87	3.06	3.07	**2.53**	**2.23**	**3.60**	2.90	**2.38**	3.36
C12	3.15	2.51	2.30	3.51	3.13	2.44	3.78	3.83	**3.10**	**2.80**	**4.53**	3.62	**3.19**	4.22
C14	11.13	9.56	9.05	12.07	10.55	9.92	11.9	11.80	**10.27**	**9.66**	**13.28**	11.36	**11.06**	12.58
C14:1c9	1.01	0.84	1.00	1.12	1.05	1.05	1.09	1.09	**0.96**	**1.02**	**1.24**	1.11	**1.20**	1.19
C16	31.04	27.16	24.26	34.38	25.78	30.01	29.31	28.79	**25.54**	**23.53**	**32.62**	26.58	**29.84**	29.33
C16:1	1.58	1.69	1.95	1.59	1.70	1.92	1.60	1.53	**1.65**	**1.83**	**1.45**	1.65	**1.72**	1.55
C17	0.64	0.66	0.72	0.61	0.70	0.66	0.70	0.64	**0.66**	**0.70**	**0.62**	0.68	**0.68**	0.66
C18	10.02	11.48	10.44	9.04	9.56	9.93	9.18	9.49	**10.66**	**10.57**	**8.36**	9.67	**8.73**	8.87
Total C18:1t	3.21	3.71	4.44	2.50	4.21	3.13	3.58	3.49	**4.01**	**4.52**	**2.97**	4.05	**3.60**	3.57
C18:1c9	19.06	23.82	26.28	16.56	21.81	23.26	17.98	21.18	**25.25**	**27.26**	**16.46**	22.62	**23.21**	19.31
Total C18:1c	20.61	25.69	28.17	17.97	23.39	25.07	19.27	22.9	**27.22**	**29.29**	**17.80**	24.36	**24.78**	20.85
Total C18:2	2.12	2.4	2.42	1.92	2.39	2.08	2.28	2.55	**2.70**	**2.8**	**2.23**	2.65	**2.33**	2.52
C18:2c9c12	1.28	1.47	1.20	1.22	1.20	1.21	1.26	1.56	**1.61**	**1.55**	**1.34**	1.5	**1.41**	1.47
C18:2c9t11	0.46	0.53	0.63	0.38	0.63	0.45	0.57	0.57	**0.62**	**0.69**	**0.52**	0.65	**0.50**	0.60
C18:3c9c12c15	0.72	0.80	1.38	0.50	1.33	0.79	1.08	0.85	**1.00**	**1.26**	**0.76**	1.12	**1.05**	0.96
SFAs	68.37	63.15	57.54	71.93	61.53	64.06	67.24	66.25	**61.39**	**58**	**71.84**	63.48	**64.81**	67.59
MUFAs	27.38	32.76	36.15	24.32	30.99	32.12	26.2	28.43	**33.08**	**36.03**	**23.24**	30.71	**30.53**	26.78
PUFAs	3.46	4.01	4.60	2.86	4.56	3.36	4.19	4.29	**4.67**	**5.11**	**3.78**	4.76	**4.17**	4.41
UFAs	30.91	36.81	40.92	27.29	35.65	35.71	30.4	32.67	**37.69**	**41.11**	**27.00**	35.41	**34.67**	31.11
SCFAs	8.71	7.92	6.88	9.06	8.24	7.35	9.04	9.12	**8.30**	**7.54**	**10.08**	8.75	**7.59**	9.46
MCFAs	50.62	44.07	41.79	55.53	45.67	48.2	51	50.44	**44.60**	**42.28**	**56.92**	48.08	**50.26**	52.81
LCFAs	39.84	47.81	49.86	34.8	43.67	44.21	38	40.64	**47.12**	**50.03**	**33.48**	43.3	**42.18**	38.01
BFAs	2.21	2.20	2.66	2.11	2.70	2.26	2.54	2.58	**2.60**	**2.77**	**2.49**	2.73	**2.60**	2.67
Omega3	0.59	0.68	0.77	0.47	0.76	0.56	0.70	0.68	**0.76**	**0.82**	**0.61**	0.77	**0.60**	0.71
Omega6	2.17	2.48	2.36	1.95	2.36	2.03	2.34	2.61	**2.73**	**2.78**	**2.30**	2.66	**2.34**	2.56
Odd-chain FAs	3.74	3.73	4.29	3.59	4.34	3.75	4.26	4.14	**4.10**	**4.35**	**4.10**	4.34	**4.41**	4.32
Total trans FAs	4.00	4.58	5.54	3.16	5.32	3.88	4.56	4.31	**4.92**	**5.62**	**3.78**	5.11	**4.33**	4.54
Total C18:1	23.85	29.4	32.28	20.64	27.24	28.27	22.49	24.76	**29.51**	**32.18**	**19.44**	26.90	**26.35**	22.94
Fat	4.11	3.94	4.01	4.23	4.10	4.05	4.25	4.17	**4.02**	**3.99**	**4.84**	4.02	**3.83**	4.15
Protein	3.44	3.31	3.43	3.50	3.5	3.37	3.55	2.55	**2.44**	**2.50**	**2.90**	2.56	**2.55**	2.65
Milk yield	26.87	26.10	22.45	28.11	24.49	24.81	25.52	30.83	**29.82**	**26.55**	**30.90**	28.56	**24.77**	30.08
EB	−2.75	−5.40	−8.50	−1.43	−7.40	−4.83	−4.04	−7.87	**−9.51**	**−10.14**	**−6.95**	−8.22	**−6.94**	−6.69
NUE	56.67	56.29	31.04	58.10	38.73	41.78	49.67	18.70	**19.76**	**19.75**	**17.96**	18.24	**20.14**	17.22
Blood BHB	−0.81	−0.74	−0.71	−0.87	−0.73	−0.77	−0.79	−0.75	**−0.67**	**−0.68**	**−0.89**	−0.73	**−0.77**	−0.80
Blood free FAs	526.9	678.60	714.60	407.70	590.40	629.00	520.30	449.3	**597.8**	**622.60**	**293.8**	514.4	**496.00**	400.8
DMI	22.24	19.93	19.78	23.51	22.03	20.84	24.17	23.49	**21.45**	**21.27**	**27.31**	23.03	**24.67**	24.49
THI	52.27	52.45	56.71	50.28	54.67	56.31	49.12	/	**/**	**/**	**/**	/	**/**	/

SFAs = saturated FAs, MUFAs = monounsaturated FAs, PUFAs = polyunsaturated FAs, UFAs = unsaturated FAs, SCFAs = short-chain FAs, MCFAs = medium-chain FAs, LCFAs = long-chain FAs, BFAs = branched FAs, EB = energy balance, NUE = Nitrogen Use Efficiency, BHB = beta-hydroxybutyrate, FFAs = free FAs, DMI = dry matter intake. THI was not calculated for the Canadian dataset, as meteorological data were not available.

**Table 8:** Percentages of organic and conventional farms in the various clusters, in % of herds present in the cluster.

	Level	Cluster 1 (*N* = 4330)	Cluster 2 (*N* = 520)	Cluster 3 (*N* = 83)	Cluster 4 (*N* = 577)	Cluster 5 (*N* = 3250)	Cluster 6 (*N* = 4)	Cluster 7 (*N* = 4639)
Farming system	Organic	8.43	11.92	3.61	12.13	8.77	0.00	8.26
	Conventional	39.33	25.77	16.87	40.56	36.15	25.00	39.21
	Unknown	52.24	62.31	79.52	47.31	55.08	75.00	52.53
Additional ventilation	No	75.83	84.75	71.43	77.91	77.19	/	75.95
	Yes	21.55	13.56	28.57	18.99	20.48	/	21.44
	Unknown	2.62	1.69	0.00	3.10	2.33	/	2.61

Data available for 2227 herds out of 4675 herds for Canada, *N* = 13,403 herds × cluster.

**Table 9:** Percentages of different feeding in the various clusters, in % of herds present in the cluster.

		Cluster 1 (*N* = 498)	Cluster 2 (*N* = 27)	Cluster 3 (*N* = 2)	Cluster 4 (*N* = 66)	Cluster 5 (*N* = 319)	Cluster 6 (*N* = 0)	Cluster 7 (*N* = 529)
Feeding	% of dry matter	50.03	31.33	0.74	49.53	46.56	/	49.83
	% of corn silage	22.44	8.63	2.04	33.98	18.22	/	22.79

Data available for 2227 herds out of 4675 herds for Canada, *N* = 13,403 herds × cluster.

**Table 10:** Means and standard deviations by cluster of management related data for 3006 Quebec herds. No observations were available for cluster 6.

Traits	Cluster 1	Cluster 2	Cluster 3	Cluster 4	Cluster 5	Cluster 7
Number of cows in lactation	65.32 ± 41.63	46.04 ± 17.1	40.55 ± 28.32	66.22 ± 34.38	58.13 ± 33.89	66.06 ± 42.37
Days in milk	174.41 ± 20.3	177.82 ± 31.35	234.75 ± 74.5	174.26 ± 19.91	176.55 ± 22.29	174.94 ± 20.31
MFEED ($CA/cow/year)	5134.67 ± 959.26	4643.00 ± 1056.39	3074.75 ± 1120.77	4932.20 ± 1056.97	5011.12 ± 1005.99	5129.53 ± 945.2
MFEED per fat yield ($CA/cow/year/kg of fat)	12.59 ± 1.17	12.83 ± 1.8	11.16 ± 0.87	12.42 ± 1.30	12.59 ± 1.35	12.58 ± 1.14
Milk yield (L/cow/day)	26.88 ± 4.58	24.61 ± 4.68	18.57 ± 5.99	25.69 ± 5.26	26.32 ± 4.66	26.83 ± 4.56
Fat (kg/cow/day)	1.08 ± 0.27	0.96 ± 0.25	0.72 ± 0.18	1.07 ± 0.28	1.04 ± 0.28	1.08 ± 0.26
Protein (kg/cow/day)	0.89 ± 0.22	0.79 ± 0.21	0.62 ± 0.19	0.87 ± 0.23	0.86 ± 0.23	0.89 ± 0.22
Milk at lactation peak (L/day)	40.09 ± 5.02	37.12 ± 5.21	32.35 ± 5.92	38.46 ± 6.35	39.39 ± 5.08	40.05 ± 5.00
Days in milk at lactation peak	44.30 ± 4.37	42.85 ± 5.92	37.5 ± 10.47	42.43 ± 4.70	44.41 ± 4.73	44.25 ± 4.34
Somatic cells (10^3^ cells/mL)	175.49 ± 106.42	183.50 ± 108.72	278.25 ± 83.43	177.64 ± 110.73	181.34 ± 111.68	177.34 ± 106.55
Somatic cells > 200,000/mL (% of cows/herd)	18.57 ± 7.44	20.80 ± 8.34	31.27 ± 9.15	19.54 ± 8.33	19.41 ± 7.52	18.69 ± 7.49
Urea (g/mL)	9.36 ± 5.33	7.47 ± 5.48	5.08 ± 5.89	9.10 ± 5.71	9.22 ± 5.59	9.42 ± 5.33
Urea < 5 or >12 g/mL (% of cows/herd)	7.50 ± 7.29	7.81 ± 7.00	6.95 ± 5.25	9.26 ± 8.72	7.98 ± 7.63	7.50 ± 7.22
Transition index	275.94 ± 406.05	53.90 ± 469.31	−406.75 ± 512.39	274.55 ± 429.99	200.7 ± 413.45	279.08 ± 401.74
Transition index < 0	35.90 ± 16.25	45.88 ± 19.16	67.5 ± 17.71	36.06 ± 17.17	38.75 ± 16.95	35.72 ± 16.05
Age at first calving (month)	24.99 ± 1.94	26.09 ± 3.27	30.15 ± 8.28	25.12 ± 2.17	25.23 ± 2.21	24.99 ± 1.92
Calving interval (days)	403.79 ± 26.51	409.22 ± 37.63	457.50 ± 95.24	404.47 ± 23.5	406.06 ± 30.01	404.24 ± 26.79
% of involuntary culling	19.43 ± 8.89	18.46 ± 8.19	20.94 ± 14.2	17.20 ± 7.79	19.83 ± 9.43	19.51 ± 8.90
% of dead cows	5.25 ± 4.63	4.78 ± 4.46	3.69 ± 4.9	5.91 ± 5.59	5.37 ± 4.94	5.33 ± 4.61
Milk MI	−289.96 ± 1223.41	−798.47 ± 1456.19	−2423.77 ± 1503.39	−358.1 ± 1256.43	−466.23 ± 1284.08	−299.49 ± 1215.2
Fat MI	−14.49 ± 52.79	−40.84 ± 62.6	−107.18 ± 55.86	−14.57 ± 55.53	−23.05 ± 54.50	−14.86 ± 52.25

MI = management index, MFEED = margin on feed costs.

## Data Availability

Restrictions apply to the availability of these data. The data were obtained from “Comité du lait” and “Lactanet” and are available upon request with the permission of the provider.

## Abbreviations

The following abbreviations are used in this manuscript:

BHB	β-hydroxybutyrate
DHI	Dairy Herd Improvement
FA	Fatty acid
FFA	Free fatty acid
FT-MIR	Fourier Transform mid-infrared
GH	Standardized Mahalanobis distance
PC	Principal component
PCA	Principal component analysis
THI	Temperature humidity index
